# The effect of *H. pylori *eradication on meal-associated changes in plasma ghrelin and leptin

**DOI:** 10.1186/1471-230X-11-37

**Published:** 2011-04-14

**Authors:** Fritz Francois, Jatin Roper, Neal Joseph, Zhiheng Pei, Aditi Chhada, Joshua R Shak, Asalia Z Olivares de Perez, Guillermo I Perez-Perez, Martin J Blaser

**Affiliations:** 1 New York University Langone Medical Center, New York, NY, USA; 2 New York Harbor Veteran Affairs Medical Center, New York, NY, USA

## Abstract

**Background:**

Appetite and energy expenditure are regulated in part by ghrelin and leptin produced in the gastric mucosa, which may be modified by *H. pylori *colonization. We prospectively evaluated the effect of *H. pylori *eradication on meal-associated changes in serum ghrelin and leptin levels, and body weight.

**Methods:**

Veterans referred for upper GI endoscopy were evaluated at baseline and ≥8 weeks after endoscopy, and *H. pylori *status and body weight were ascertained. During the first visit in all subjects, and during subsequent visits in the initially *H. pylori*-positive subjects and controls, blood was collected after an overnight fast and 1 h after a standard high protein meal, and levels of eight hormones determined.

**Results:**

Of 92 enrolled subjects, 38 were *H. pylori*-negative, 44 *H. pylori*-positive, and 10 were indeterminate. Among 23 *H. pylori*-positive subjects who completed evaluation after treatment, 21 were eradicated, and 2 failed eradication. After a median of seven months following eradication, six hormones related to energy homeostasis showed no significant differences, but post-prandial acylated ghrelin levels were nearly six-fold higher than pre-eradication (p = 0.005), and median integrated leptin levels also increased (20%) significantly (p < 0.001). BMI significantly increased (5 ± 2%; p = 0.008) over 18 months in the initially *H. pylori*-positive individuals, but was not significantly changed in those who were *H. pylori*-negative or indeterminant at baseline.

**Conclusions:**

Circulating meal-associated leptin and ghrelin levels and BMI changed significantly after *H. pylori *eradication, providing direct evidence that *H. pylori *colonization is involved in ghrelin and leptin regulation, with consequent effects on body morphometry.

## Background

The healthful regulation of energy homeostasis in humans, depends on centrally-acting hormones such as ghrelin and leptin [1,2]. Serum ghrelin concentrations increase during fasting, and decrease after eating [3]; ghrelin decreases energy expenditure and promotes weight gain [4]. In contrast, leptin produced primarily by adipocytes, reduces appetite and increases energy utilization [5]. The gastric epithelium expresses both ghrelin and leptin (and their receptors) [6,7]; inflammation can modify their production [8,9].

*Helicobacter pylori*, which colonizes the human stomach and interacts with host tissues [10] may affect the regulation of ghrelin and leptin [9]. However, ghrelin and leptin expression in *H. pylori*-colonized hosts has been reported as reduced [11], or increased. Similarly, body mass index (BMI) has been reported to be increased [12] or reduced [13] following *H. pylori *eradication.

We hypothesized that gastric *H. pylori *colonization affects the physiologic regulation of gut hormones involved in food intake, energy expenditure, and body weight maintenance. The hormones that affect overall metabolic function include ghrelin, leptin, amylin, insulin, active glucagon-like peptide-1, gastric inhibitory polypeptide, peptide YY, and pancreatic polypeptide. We used clinically indicated *H. pylori *eradication to evaluate the effect of *H. pylori *on meal-associated changes in ghrelin, leptin, and the other specified insulinotropic and digestive hormones, and to assess post-eradication changes in body mass index.

## Methods

### Study population

Adults ≥ 18 years of age undergoing routine upper endoscopy for any indication at the ambulatory endoscopy unit at the New York campus of the VA New York Harbor Healthcare System were prospectively recruited, as described [9,14]. The Institutional Review Board approved the study protocol, and written informed consent was obtained from all participants.

### Clinical evaluation and specimen collection

Each patient had a history and physical examination, and fasted for 12-hours overnight prior to the endoscopy. Demographic and clinical information, including assessment of dyspeptic symptoms (Additional file 1), was collected via a standardized questionnaire administered by trained interviewers at the time of study entry. Ethnic designation was self-reported by participants as White Non-Hispanic, Black Non-Hispanic, Hispanic, or Asian. Participants wore light clothing without shoes; height and weight were measured using the same column scale with a telescopic height rod, and BMI calculated. Between 8 am and 10 am, 15 ml of blood was collected in EDTA-coated tubes from the fasting patients prior to endoscopy. All samples were centrifuged, and stored as serum at -20 C, until examined.

### Endoscopy

Complete endoscopic evaluation of the upper gastrointestinal tract was performed in standard fashion to the 2^nd ^portion of the duodenum, after intravenous administration of meperidine and midazolam, as described [14]. Gastric inflammation was graded using the Sydney-Houston system [15]. Using standard forceps, two biopsies each were obtained from the gastric antrum, body, and fundus in accordance with the updated Sydney classification; two additional antral biopsies were used for rapid urease testing.

### Histological analysis

One biopsy specimen from each of the three sites was fixed in 10% formalin, embedded in paraffin, and 5 μm consecutive sections obtained for histologic staining. A single experienced GI pathologist (Z.P.) blinded to the data, graded the extent of gastritis and intestinal metaplasia on a scale of 0 to 3+ according to the Sydney classification [16]. Active gastritis refers to the presence of neutrophils in the histopathology, and chronic active reflects both neutrophils and mononuclear cells. For active gastritis, total score < 2 was defined as "low" while ≥ 2 was defined as "high". *H. pylori *was detected using the cresyl violet stain for the identification of spiral or curved-shaped organisms near the mucous layer [17].

### *H. pylori *status determination

Along with histologic evaluation and the rapid urease assays, *H. pylori *was assessed using one other tissue-based method (bacteriologic culture, as described) [18], and by two serologic methods. Serum samples were examined by ELISA for IgG antibodies to *H. pylori *whole cell and CagA antigens, with results expressed as OD ratios relative to laboratory standards, as described [19,20]. Subjects were considered *H. pylori*-positive if positive by histologic examination or culture, or if positive by rapid urease assay, and by IgG antibodies to *H. pylori *group or CagA antigens. Subjects were considered indeterminate for *H. pylori *if only the rapid urease test or one serological test was positive, consistent with prior studies [9].

### Test meal

Following recovery from endoscopy-related sedation, all patients ate a high-protein non-commercial meal averaging 806 calories. The contents of the meal were selected with the guidance of a trained nutritionist to provide 72 g carbohydrate, 71 g protein, and 26 g fat. Given that the trough that occurs in serum ghrelin levels occurs one hour postprandially [3], 15 ml of blood was collected one hour after completing the meal, and processed as above.

### *H. pylori *eradication therapy

Patients who tested positive for *H. pylori *were offered a 14-day twice-daily regimen [amoxicillin 1000 mg, clarithromycin 500 mg, and a proton pump inhibitor (PPI; omeprazole 20 mg, rabeprazole or esomeprazole 40 mg)] [21]. Seven patients who had two positive serologic tests were treated to eradicate *H. pylori *after further confirmation with the ^13^C Urea Breath Test while off antisecretory medications. One penicillin-allergic patient received metronidazole [500 mg twice a day] instead of amoxicillin. In accordance with current guidelines [22], *H. pylori *eradication was ascertained using the ^13^C Urea Breath Test, according to the manufacturer's instructions (Meretek Diagnostics, Rockville, MD) ≥8 weeks after treatment ended. At that time blood (15 ml) was again collected after fasting and 1 hour after a standardized meal. Patients who failed eradication were treated for 14 days with bismuth subsalicylate (525 mg four times a day), combined with a twice daily regimen [tetracycline (500 mg), metronidazole (500 mg), and PPI] [21]. The ^13^C Urea Breath Test was repeated in all patients who completed rescue therapy. If not clinically indicated, antisecretory medications were not continued beyond the treatment period necessary for eradication.

### Metabolic tests

A multi-hormone EIA panel (Catalogue HGT-68K; Millipore Corp., Billerica MA) was used to quantify eight gut hormones that are important regulators of food intake [1,3,23], energy expenditure [24], and body weight [25] via the gut-brain axis: acylated (active) ghrelin, leptin, active amylin, insulin, active glucagon-like peptide-1 (GLP-1), total gastric inhibitory polypeptide (GIP), total peptide YY (PYY), and pancreatic polypeptide (PP). We have previously reported a significant correlation between this assay and a standard enzyme linked immunoassay (EIA) for leptin (r = 0.57, p = 0.004). Similarly, we have found the correlation with a standard ghrelin EIA to be significant [r = 0.37; p = 0.018 (data not shown)]. The intra- and inter-assay variabilities ranged between11% and 19%, respectively, according to the manufacturer, and for the ghrelin assay no cross reactivity exists with desacyl ghrelin. All tests were performed in duplicate on coded samples.

### Statistical analysis

Continuous variables were compared using the *t*-test, or ANOVA method, and pair-wise analyses (e.g. pre-meal vs. post-meal, baseline vs. eradicated) were performed using non-parametric tests (Wilcoxon's signed rank test, Mann-Whitney U test), as appropriate. Data are expressed as mean ± SD, or median and interquartile range (25^th^- 75^th ^percentile). Categorical variables were compared using the Chi-squared test with Yates' correction or using Fisher's exact test. Spearman correlation coefficients were calculated for the relationship of leptin and ghrelin to BMI. Corrections were made in instances of multiple comparisons using techniques such as Tukey's range test. Based on previous findings showing 75% increase in fasting ghrelin after *H. pylori *eradication [13], our study was powered to allow for the detection of at least a 30% difference in ghrelin levels following successful eradication of 15 patients. Statistical analysis was performed using SPSS software version 16.0 for Macintosh (SPSS Inc., Chicago, Illinois); a two-tailed p-value of < 0.05 was considered significant.

## Results

### Patient demographic and clinical characteristics

We enrolled 92 patients who completed the test meal protocol, as shown in Additional file 2, Figure S1. Based on histologic, culture, and serologic results, 38 patients were categorized as *H. pylori*-negative, 44 as *H. pylori*-positive, and 10 were indeterminate (Table 1). Compared to *H. pylori*-negative patients baseline BMI was significantly higher among *H. pylori*-positive patients. The prevalence of diabetes was also higher among *H. pylori*-positive compared to *H. pylori*-negative patients, however this difference did not reach statistical significance. The most common indication for endoscopy in both the *H. pylori*-negative and *H. pylori*-positive patients was heme-positive stool, and persistent heartburn in the *H. pylori*-indeterminate group (shown in Additional file 3, Table S1); PPI use occurred in 46% of the entire study group. Endoscopic findings did not differ significantly between the groups (Additional file 3, Table S2). There were no significant changes in the maintenance use of antisecretory medications between baseline and follow-up examinations. We excluded the *H. pylori*-indeterminate group from subsequent analyses.

**Table 1:** Demographic and clinical characteristics of the 92 study patients, according to *H. pylori *status

Characteristic	*H. pylori*-negative (N = 38)	*H. pylori*-indeterminate (N = 10)	*H. pylori*-positive (N = 44)	Comparison of *H. pylori*-negative and *H. pylori*-positive subjects (p-value)
Mean age (years) ± SD	65 ± 13	70 ± 6	64 ± 14	0.84^b^

Male, n (%)	36 (95)	10 (100)	43 (98)	0.47^c^

Race/ethnicity, n (%)				0.22^c^
White, non-Hispanic	19 (50)	4 (40)	13 (30)	
Black, non-Hispanic	13 (34)	3 (30)	18 (41)	
Hispanic	5 (13)	3 (30)	12 (27)	
Asian	1 (3)	0 (0)	1 (2)	

Mean BMI (kg/m^2^) ± SD	26.4 ± 4	26.0 ± 3	29.4 ± 5	0.008^b^

PPI use, n (%)^a^	15 (40)	9 (90)	18 (41)	0.54^c^

Diabetes, n (%)	7 (18)	3 (30)	14 (32)	0.17^c^

^a ^PPI = proton pump inhibitor

^b ^Student's *t*-test; ^c ^Chi-Squared test

The *H. pylori*-negative and -positive groups did not differ significantly in age, ethnicity, PPI use, gender, or prevalence of upper abdominal symptoms, (Table 1), but as expected, they differed in extent of acute gastritis (Additional file 3, Table S3). At baseline, the *H. pylori*-negative subjects had lower BMI measurements than did the *H. pylori*-positive group (26.4 ± 4 vs. 29.4 ± 5; p = 0.008). Stratifying the 44 *H. pylori*-positive hosts according to *cagA *status of their strain did not reveal any significant differences in baseline demographic and clinical parameters (data not shown).

### Energy homeostasis hormones

The study subjects varied substantially in baseline pre-meal (fasting) serum values for the eight studied hormones (Additional file 3, Table S4). As expected, serum leptin values correlated with BMI, for both the *H. pylori*-negative and *H. pylori*-positive subjects (Additional file 2, Figure S2). There were no significant differences according to *H. pylori *status in pre-meal leptin, amylin, insulin, ghrelin, GIP, GLP-1, PP, and PYY levels (Additional file 3, Table S4). As expected, there were hormonal responses to the test meal; post-meal amylin levels rose physiologically [26] in both the *H. pylori*-negative and *H. pylori*-positive subjects (Additional file 3, Table S4). Similarly, there were significant post-meal increases in the levels of insulin, GIP, PP, and PYY, in both the *H. pylori*-negative and *H. pylori*-positive groups. As expected [3], ghrelin values diminished following the meal, while leptin values rose significantly in both groups. Thus, our observations are consistent with the expected meal-associated hormonal changes, with no significant differences between the *H. pylori*-positive and -negative subjects, as well as when data were normalized (Additional file 3, Table S5).

### Effects of *H. pylori *eradication

**Figure 1:**
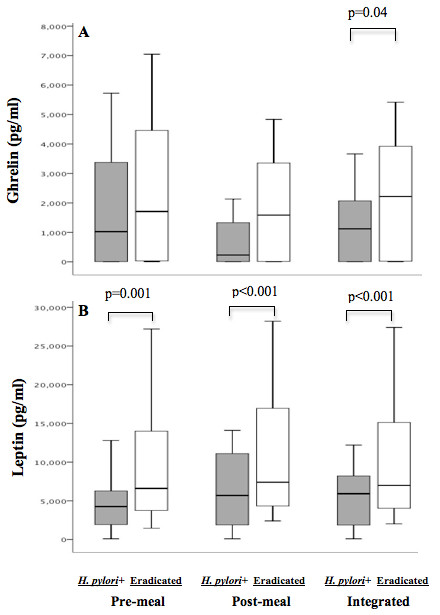
**Comparison of *H. pylori*+ persons at baseline, and then after eradication of *H. pylori***. A standardized meal was administered to 21 subjects, and pre-meal, post-meal, and integrated values (mean of pre-meal and post-meal) were calculated for acyl-ghrelin (**Panel A**) and leptin (**Panel B**). *H. pylori+*(grey), Eradicated (white). Boxes indicate median and interquartile range, and bars indicate minimum and maximum values. P-values represent significant (< 0.05) differences between the *H. pylori^+ ^*and post-eradication samples.

Treatment for *H. pylori *was accepted by 31 (70.5%) of the 44 subjects in whom it was clinically indicated; 23 completed all of our assessments and eradication was successful in 21 (91%) (Additional file 2, Figure S1). The 21 subjects were representative of the entire group of 44 who were initially *H. pylori*-positive, with similar meal-associated hormone changes at baseline (compare Table 2, and Additional file 3, Table S4). Following *H. pylori *eradication, the meal-associated increases in amylin, insulin, GIP, PP, and PYY remained significant (Table 2). Compared to baseline, post-meal levels of the incretin GLP-1 were significantly increased following *H. pylori *eradication. Pre-meal ghrelin levels did not significantly differ between baseline and post-eradication (Table 2); however, following *H. pylori *eradication, post-meal ghrelin levels did not substantially decrease (Figure 1A). After *H. pylori *eradication, pre-meal, post-meal, and integrated leptin levels rose significantly (Figure 1B), and remained significantly correlated with BMI (r = 0.69, p < 0.01). PPI use did not account for the changes in ghrelin and leptin levels from baseline to follow-up (data not shown). This finding is consistent with other reports that PPI use does not influence ghrelin levels[27,28].

**Table 2:** Levels of eight hormones related to energy homeostasis, and BMI in 21 subjects, according to *H. pylori *eradication status

	Median (IQR) hormone concentration (pg/ml), before and after *H. pylori *eradication						Comparison of values at baseline and after eradication (p-value)	
	**Baseline**			**Eradicated**				

**Hormone**	**Pre-meal**	**Post-meal**	**p^b^**	**Pre-meal**	**Post-meal**	**p^b^**	**Pre-meal**	**Post-meal**

Amylin	15	19	**0.046**	16	36	**0.008**	0.25	0.16
	(14-43)	(15-54)		(15-50)	(15-106)			

Insulin	243	584	**< 0.001**	265	763	**< 0.001**	0.39	0.28
	(129-414)	(462-1,475)		(139-558)	(430-2,100)			

Ghrelin	1,024	231	**0.004**	1,710	1,586	0.12	0.51	**0.005**
	(7-3,461)	(7-1,329)		(27-4,573)	(13-3,360)			

GIP	15	107	**0.001**	27	82	**0.001**	0.82	0.85
	(5-29)	(29-214)		(9-54)	(41-282)			

GLP-1	28	16	0.51	36	45	0.95	0.11	**0.04**
	(10-56)	(9-59)		(12-89)	(14-91)			

Leptin	4,260	5,690	**0.02**	6,605	7,400	**0.02**	**0.001**	**< 0.001**
	(1,890-6,649)	(1,765-11,350)		(3,517-14,600)	(4,120-17,925)			

PP	63	107	**0.005**	79	112	**0.001**	0.31	0.23
	(18-113)	(44-204)		(26-153)	(45-172)			

PYY	39	70	**0.002**	61	70	**0.001**	**0.02**	0.34
	(27-82)	(48-98)		(34-90)	(47-137)			

^a ^p-values < 0.05 are indicated in **bold**

^b ^Wilcoxon's signed rank test comparing pre-meal and post-meal values.

^c ^Paired *t*-test comparing BMI at baseline to after *H. pylori *eradication.

**Figure 2:**
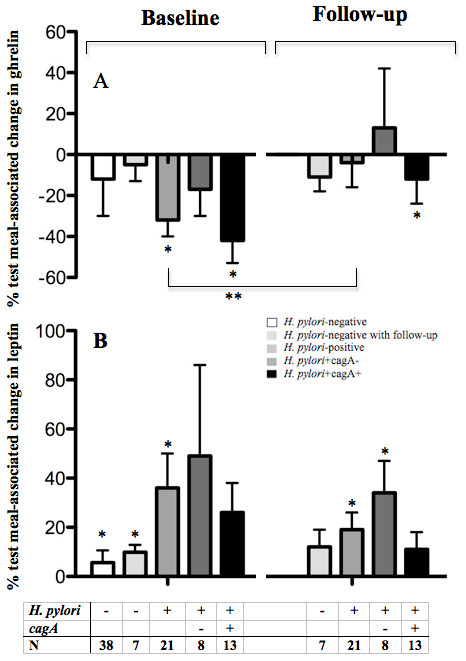
**Comparison of test-meal induced changes in plasma acyl-ghrelin and leptin levels according to *H. pylori *at baseline and after eradication**. Data are for seven *H. pylori*-negative, 21 *H. pylori*-positive subjects including eight *H. pylori+*cagA- and 13 *H. pylori*+cagA+ subjects. (* P < 0.05, comparing either pre-meal to post-meal values, or **comparing the first and second evaluations). Data also are shown for all of the 38 *H. pylori*-negative subjects at baseline, for comparison with the subset who also had follow-up studies. Panel A: Ghrelin levels. Panel B: Leptin Levels.

Since the initial measurements were performed on the day of endoscopy while the second measurements were not, we considered that gastric distension might have influenced measurements between the two time-points. We addressed this potential bias by also evaluating seven subjects who were *H. pylori *negative at a second time-point. The same test meal, and metabolic evaluations were repeated in the seven *H. pylori*-negative subjects at baseline and after follow-up (median 14 months). As expected, this control group had no significant changes in the eight measured adipokines between the two time-points (data not shown), and no changes in meal-associated physiology (Figure 2). In the *H. pylori*-positive subjects at baseline, ingestion of the test meal led to a 32 ± 9% decrease (p = 0.004) in ghrelin with somewhat larger declines in persons with *cagA*-positive strains than in those with *cagA*-negative strains. Data from the 38 *H. pylori*-negative subjects and the subset of seven who had long-term follow-up also showed similar trends (Figure 2A). However, after *H. pylori *eradication, post-meal ghrelin levels only fell minimally (4 ± 12%; p = NS); the difference in meal-associated responses comparing baseline and post-eradication (32% vs. 4%) was significant (p = 0.05). At baseline, leptin levels in both the *H. pylori*-positive and *H. pylori*-negative subjects significantly increased after the test meal (Figure 2B). The meal-associated rise in leptin after eradication (19 ± 7%), remained significant (p = 0.02). Following *H. pylori*-eradication in subjects previously colonized with *cagA*-positive strains, the expected meal-associated increase in PP was significantly lower than expected (27% vs. -13%; p = 0.01).

### Meal-associated ghrelin physiology in relation to baseline gastric histology

In the group from whom *H. pylori *was eradicated, the severity of histologic inflammation in the fundus at baseline was negatively correlated with pre-meal ghrelin (r = -0.57, p = 0.01), as expected [29,30]. Subjects with more active gastritis had higher pre-meal ghrelin levels at baseline, and greater meal-associated changes post-eradication (Additional file 3, Table S6). Eradication-related changes in ghrelin physiology also correlated with the anatomic location of gastritis; subjects with antral gastritis only showed the largest increases in values obtained pre-meal, post-meal, and across the meal (Additional file 3, Table S7). These data provide evidence that both location and extent of gastric inflammation affects ghrelin secretion, but similar associations were not found with reference to leptin physiology (data not shown).

### Body mass index in relation to *H. pylori *status

**Figure 3:**
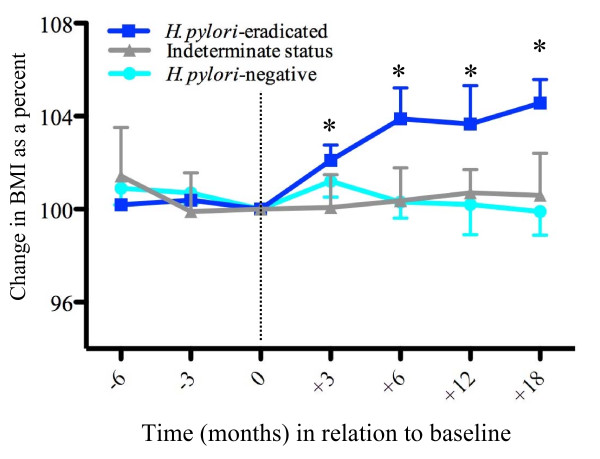
**Change in BMI in 69 study subjects over a 2-year period**. BMI is calculated relative to the baseline (at time 0), and is shown from 6 months prior to baseline and during 18 months of follow-up in 38 *H. pylori*-negative, 21 *H. pylori*-eradicated, and 10 subjects who were *H. pylori*-indeterminate at baseline (*p < 0.05, comparing time 0 to other follow-up months).

Since baseline BMI was higher for the *H. pylori*-eradicated group, we addressed this potential bias by comparing individuals to themselves in longitudinal pair-wise analyses. During the six months prior to study initiation, BMI did not change substantially in any of the study subjects (Figure 3). During 18 months (IQR 12, 24) of follow-up, BMI did not change significantly in subjects who at baseline were either *H. pylori*-negative or *H. pylori*-indeterminate. In contrast, in the *H. pylori*-eradicated group, BMI progressively and significantly increased, reaching 105 ± 2% by 18 months of follow-up (p = 0.008); baseline *H. pylori cagA *status did not predict results (p = 0.58). The change in BMI relative to baseline also was significantly greater at 3, 6, and 12 months following eradication compared to the *H. pylori*-negative group (data not shown). The change in pre-meal ghrelin from baseline following *H. pylori *eradication, was positively correlated with the change in BMI at 3 months (r = 0.78; p = 0.005), 6 months (r = 0.86; p = 0.001), and 12 months [r = 0.82; p = 0.001 (Additional file 2, Figure S3)], and 18 months (r = 0.87; p = 0.001).

### Dyspepsia symptoms at baseline and follow-up

Since weight gain could reflect decreased dyspeptic symptoms following *H. pylori *eradication, we assessed dyspeptic symptoms at baseline and post-eradication. A validated multidimensional assessment tool [31] was used to evaluate three scales: pain intensity, non-pain symptoms, and satisfaction with dyspepsia-related health. At baseline, the 38 *H. pylori*-negative and 44 *H. pylori*-positive subjects did not differ significantly in median pain, non-pain, and satisfaction scores (data not shown). Among the 21 patients from whom *H. pylori *was eradicated, there were no significant differences between baseline and follow-up pain scores [Median (IQR) 9 (2-23) vs. 6 (2-15); p = 0.86], non-pain scores [13 (12-16) vs. 10 (10-18); p = 0.28], or satisfaction scores [13 (10-23) vs. 19 (12-20); p = 0.29]. Thus, the observed increase in BMI following eradication (Figure 3) was not correlated with diminished dyspepsia that could increase appetite.

## Discussion

Appetite-reducing hormones, such as amylin, insulin, GIP, GLP-1, PP, and PYY, produced in the small intestine and pancreas are important in mammalian energy homeostasis, [32-35], as is leptin which is produced mostly by adipocytes, but also by gastric chief cells [6]. Importantly, gastric oxyntic endocrine cells [36] account for 65-80% of the body's total ghrelin production. *H. pylori *colonization status has been correlated with circulating and gastric mucosal leptin levels [9], and with gastric mRNA expression and plasma levels of ghrelin [9,37]. We now identified substantial effects of *H. pylori *eradication on meal-associated changes in gastric hormones and energy balance, confirming and extending prior studies in a more rigorous manner [13,38].

That *H. pylori*-positive and *H. pylori*-negative subjects had similar baseline digestive hormonal physiologies (Additional file 3, Table S4) may reflect the highly integrated cross-regulation of energy homeostasis, [39] and the long-term equilibria between *H. pylori *and individual hosts [10,40]. Our observations of high pre-meal levels of acylated (acyl-) ghrelin that then fell post-prandially were as expected [3]. However, several months after *H. pylori *eradication, the extent of physiologic meal-associated reduction in circulating acyl-ghrelin was much diminished. These findings are consistent with other studies of subjects who underwent *H. pylori *eradication and then had increased plasma ghrelin [13,30,38] and gastric ghrelin mRNA levels [29]. Alterations in ghrelin regulation following *H. pylori *eradication may reflect the extent of baseline gastric inflammation [8]. Similarly, plasma levels of acyl-ghrelin may be significantly elevated post-*H. pylori *eradication, and vary reflecting the severity of atrophic gastritis [30], but atrophic gastritis is uncommon in the population we studied (data not shown). Methodological issues across studies, such as the length of follow-up post-*H. pylori *treatment [11,29] and differences in populations examined [11] may partially account for differing metabolic and anthropometric findings. We now provide evidence that the extent and location of *H. pylori*-induced inflammation at baseline is associated with the differences in ghrelin physiology that develop due to *H. pylori *eradication. Although our data must be considered preliminary with small numbers of subjects, baseline antral gastritis appears to affect responses to eradication.

Although ghrelin is known to induce weight gain, in a study with six weeks of follow-up after *H. pylori *eradication, plasma ghrelin was increased, but median BMI was unchanged [13]. In another study, 12 weeks following *H. pylori *eradication, plasma ghrelin was increased in some subjects and reduced in others [29].

Our study now shows that following *H. pylori *eradication, there is blunting of the meal-associated physiologic reduction in circulating acyl-ghrelin, and there is long-term weight gain; in addition, changes in baseline acyl-ghrelin values and changes in BMI were linked (Additional file 2, Figure S3). Reflecting the observed weight gain, leptin levels pre-meal and post-meal ghrelin levels were significantly elevated after eradication and differed significantly from baseline values. We also observed that *H. pylori *eradication was associated with preservation of the expected [26,41-43] meal-associated increases in amylin, insulin, GIP, PP, and PYY. Post-meal levels of the incretin GLP-1 were significantly increased following eradication compared to baseline, perhaps reflecting the need for a meal-termination signal in the setting of persistently elevated ghrelin levels. We found no evidence that the weight gain associated with *H. pylori *eradication reflected improvement of dyspeptic symptoms as suggested previously [44]. Although in our study group, the *H. pylori*-positive subjects had higher BMIs at baseline compared to *H. pylori*-negative, the study was not designed to compare BMI between *H. pylori*-negative and *H. pylori*-positive groups. Rather, we sought to compare the change in BMI over time within the groups.

Our findings are limited by the study setting at a veteran's hospital where most of the evaluated patients were older men. Measurement of ghrelin is not standardized, [45] and may account for the substantial inter-individual variation that we report. However, comparing each subject to himself before and after the standard test meal, and repeating the same measurements at baseline and during follow-up reduces the effects of inter-individual variation, as well as any potential effect of the endoscopic evaluation performed on all patients. We also verified measurements in the same individuals in duplicate on separate occasions. Since all patients (*H. pylori*-negative and *H. pylori*-positive) had an endoscopic evaluation prior to the first postprandial measurement of hormones, and not prior to the second postprandial measurement, the observed post-eradication changes could not be explained by the potential effect of the endoscopic examination alone. We measured acyl-ghrelin, which also may be relevant in energy homeostasis following *H. pylori *eradication, as opposed to total ghrelin as others have done [11,29]. We did not address changes in the ratio of circulating active versus inactive ghrelin before and after eradication. Other strengths of the study include the collection of detailed demographic, clinical, and histologic data from a prospectively enrolled group using validated instruments, *H. pylori *status determination using multiple methods for all patients that improve sensitivity and diminish falsely negative categorization [46], measurement of eight gut hormones to ascertain the meal-associated metabolic profile of each subject at baseline, and following up an *H. pylori*-negative group for comparison. We planned to analyze patients who were not successfully treated to eradicate *H. pylori*, however few individuals failed eradication therapy and thus that control group was not sufficiently populated. In addition, our ability to further analyze *H. pylori*-negative patients was limited by the fact that only 7 completed follow-up evaluations.

## Conclusions

In conclusion, our study indicates that leptin and ghrelin physiology change and that BMI increases following *H. pylori *eradication. Although the number of subjects is limited, using patients as their own controls and having multiple measurements allowed us to both confirm previous published data, and to use a standard meal technique to extend the findings. This study provides further evidence that gastric *H. pylori *is involved in the physiologic regulation of these hormones, and supports the rationale for randomized controlled *H. pylori *eradication trials to focus on the role of inflammation and endocrine cross-talk in explaining these findings.

## Competing interests

The authors declare that they have no competing interests.

## Authors' contributions

All authors read and approved the final manuscript. FF participated in the design of the study, patient recruitment, sample procurement, statistical analysis, and manuscript preparation. JR participated in patient recruitment, sample processing, and manuscript preparation. NJ contributed with patient recruitment and with manuscript preparation. ZP was involved in review of pathology samples and manuscript. AC participated in patient recruitment and manuscript review. JRS participated in sample processing and manuscript preparation. AZO was involved in sample procurement and processing as well as manuscript review. GIP participated in sample processing and manuscript preparation. MJB participated in study design, analysis, and manuscript preparation.

## Pre-publication history

The pre-publication history for this paper can be accessed here:

http://www.biomedcentral.com/1471-230X/11/37/prepub

## Supplementary Material

Additional file 1 **Supplemental methods**. Provides information regarding exclusion criteria for the study population, symptom evaluation, *H. pylori *evaluation, test meal, and metabolic tests. Click here for file

Additional file 2 **Supplemental figures**. Provides information regarding the enrollment and classification of study participants (Figure S1), the relationship of baseline BMI and baseline pre-meal leptin according to *H. pylori *status (Figure S2), and the correlation of changes in ghrelin and BMI post-*H. pylori *eradication (Figure S3). Click here for file

Additional file 3 **Supplemental tables**. Provides information regarding the indications for upper GI endoscopy among study subjects (Table S1), findings during upper endoscopy (Table S2), Histologic score at three gastric sites according to *H. pylori *status (Table S3), levels of eight hormones related to energy homeostasis at the baseline evaluation of subjects according to *H. pylori *status and in relation to the test meal (Table S4), test-meal induced change and normalized change in hormone profile according to *H. pylori *status at baseline (Table S5), comparison of baseline and post-eradication meal-associated ghrelin profile in 21 originally *H. pylori*-positive subjects according to severity of baseline histologic gastritis (Table S6), and meal-associated changes in ghrelin profile in 28 subjects^a ^who had follow-up evaluation according to anatomical distribution of histologic gastritis at baseline (Table S7). Click here for file
