# Supplementary material for: The effect of H. pylori eradication on meal-associated changes in plasma ghrelin and leptin
Source: BMC Gastroenterol. 2011 Apr 14;11:37. doi: 10.1186/1471-230X-11-37 (PMC3089783; doi:10.1186/1471-230X-11-37)
Supplement: Additional file 1 — Supplemental methods. Provides information regarding exclusion criteria for the study population, symptom evaluation, H. pylori evaluation, test meal, and metabolic tests. [file 1471-230X-11-37-S1.DOC]

**SUPPLEMENTAL METHODS:**

**Study population.**  Participants were excluded if they had a history of esophageal or gastric varices, prior gastric surgery, known coagulopathy, had treatment with corticosteroids or other immunomodulating drugs in the month prior to enrollment, or if they had been treated to eradicate *H. pylori*

Symptom evaluation. Clinical gastroesophageal reflux disease (GERD) was defined as the presence of heartburn, regurgitation, or dysphagia [47], occurring at least weekly in the 4 weeks prior to enrollment. A standardized questionnaire was used to grade each symptom on a scale of 0 to 3. Dyspepsia was defined as the presence of chronic or recurrent abdominal pain in the center of the upper abdomen . The validated Severity of Dyspepsia Assessment (S.O.D.A.) questionnaire [31] was used to grade pain intensity, non-pain symptoms, and general satisfaction before and after treatment to eradicate *H. pylori*.

***H. pylori* status determination.** For culture, gastric biopsies were placed immediately in normal saline at 4^o^ C and coarsely homogenized in 250μl of normal saline. From this suspension, 50-μl aliquots were inoculated onto trypti­case soy agar plates with 5% sheep blood, 50μl of Skirrows medium and incubated for 96 h under microaerobic conditions, as described. *H. pylori* cells were identified as pleiomorphic, curved, gram-negative bacilli that were oxidase, urease, and catalase positive.

**Test meal.** Each food tray contained 1 hard boiled egg, 35g of cereal with raisins, 237ml Ensure (Abbott laboratories, Abbott Park, IL), 236ml cranberry juice, 236ml 2% milk, 30ml sugar-free protein supplement, 116ml coffee.

**Metabolic tests.** An enzyme-linked immunosorbent assay (R&D Systems, Minneapolis MN) also was used to measure serum leptin levels, as described. The precision of the leptin assay is 94.6-96.5% and the minimum detectable level is 7.8 pg/ml [9, 14]. The leptin concentrations obtained using the multi-hormone panel and the specific EIA were correlated, with r=0.84 (p<0.001).
