# Supplementary material for: The effect of H. pylori eradication on meal-associated changes in plasma ghrelin and leptin
Source: BMC Gastroenterol. 2011 Apr 14;11:37. doi: 10.1186/1471-230X-11-37 (PMC3089783; doi:10.1186/1471-230X-11-37)
Supplement: Additional file 2 — Supplemental figures. Provides information regarding the enrollment and classification of study participants (Figure S1), the relationship of baseline BMI and baseline pre-meal leptin according to H. pylori status (Figure S2), and the correlation of changes in ghrelin and BMI post-H. pylori eradication (Figure S3). [file 1471-230X-11-37-S2.DOC]

**Figure S1. Enrollment and classification of 92 study participants**. All study participants had an initial metabolic evaluation, and a second evaluation was provided to those who completed eradication therapy. The second evaluation also was offered to those who were initially negative, to serve as a control group.

**Figure S2. Relationship of baseline BMI and baseline pre-meal leptin in 38 *H. pylori*-negative (**🌣**)** **and 44 *H. pylori*-positive** **(**🛆**)** **subjects**. Correlation is by linear regression analysis (*H. pylori*-negative r=0.55, p<0.001; *H. pylori*-positive r=0.60, p<0.001).

**Figure S3. Correlation of changes in ghrelin and BMI post-*H. pylori* eradication**. The ordinate shows the log_10_ percent change in pre-meal ghrelin associated with eradication, and the abscissa shows changes in BMI at 12 months, relative to baseline in 17 *H. pylori*-eradicated subjects (r=0.82, p<0.001).

**Figure S1.**

Patients enrolled

(n = 92)

Not treated

(n = 13)

Treated

(n = 31)

*H. pylori*-not eradicated

(n = 2)

*H. pylori*-eradicated

(n = 21)

Follow-up evaluation

(n = 23)

Follow-up pending

(n = 8)

Follow-up evaluation

(n = 7)

*H. pylori*-positive

(n = 44)

*H. pylori*-negative

(n = 38)

*H. pylori*-indeterminate

(n = 10)

**Metabolic Evaluation 1**

**Metabolic Evaluation 2**

**Figure S2.**

**
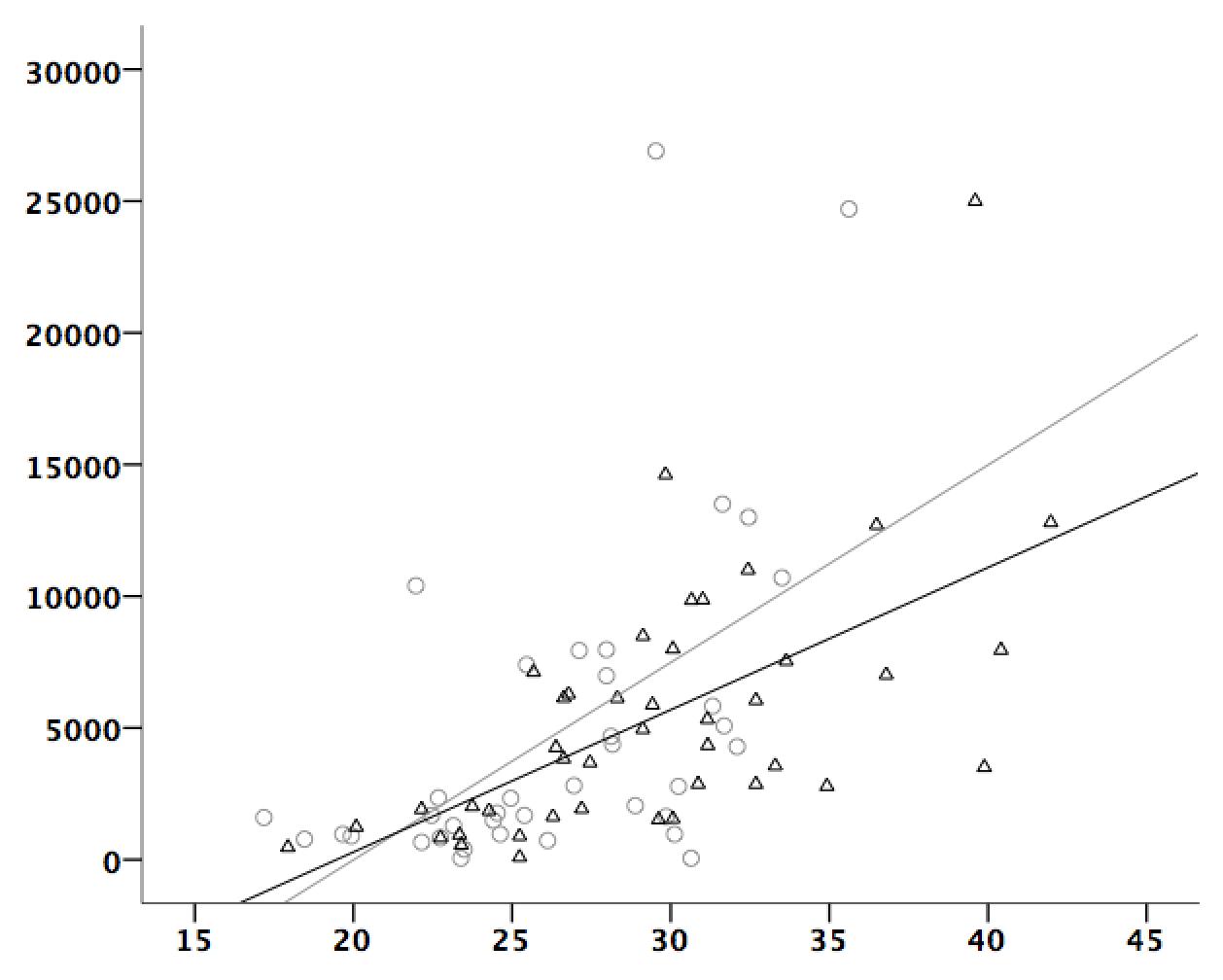
**

Serum leptin (pg/ml)

🌣*H. pylori*-negative r=0.55; p<0.001

🛆 *H. pylori*-positive r=0.60; p<0.001

Body Mass Index (kg/m^2^)

**Figure S3.**


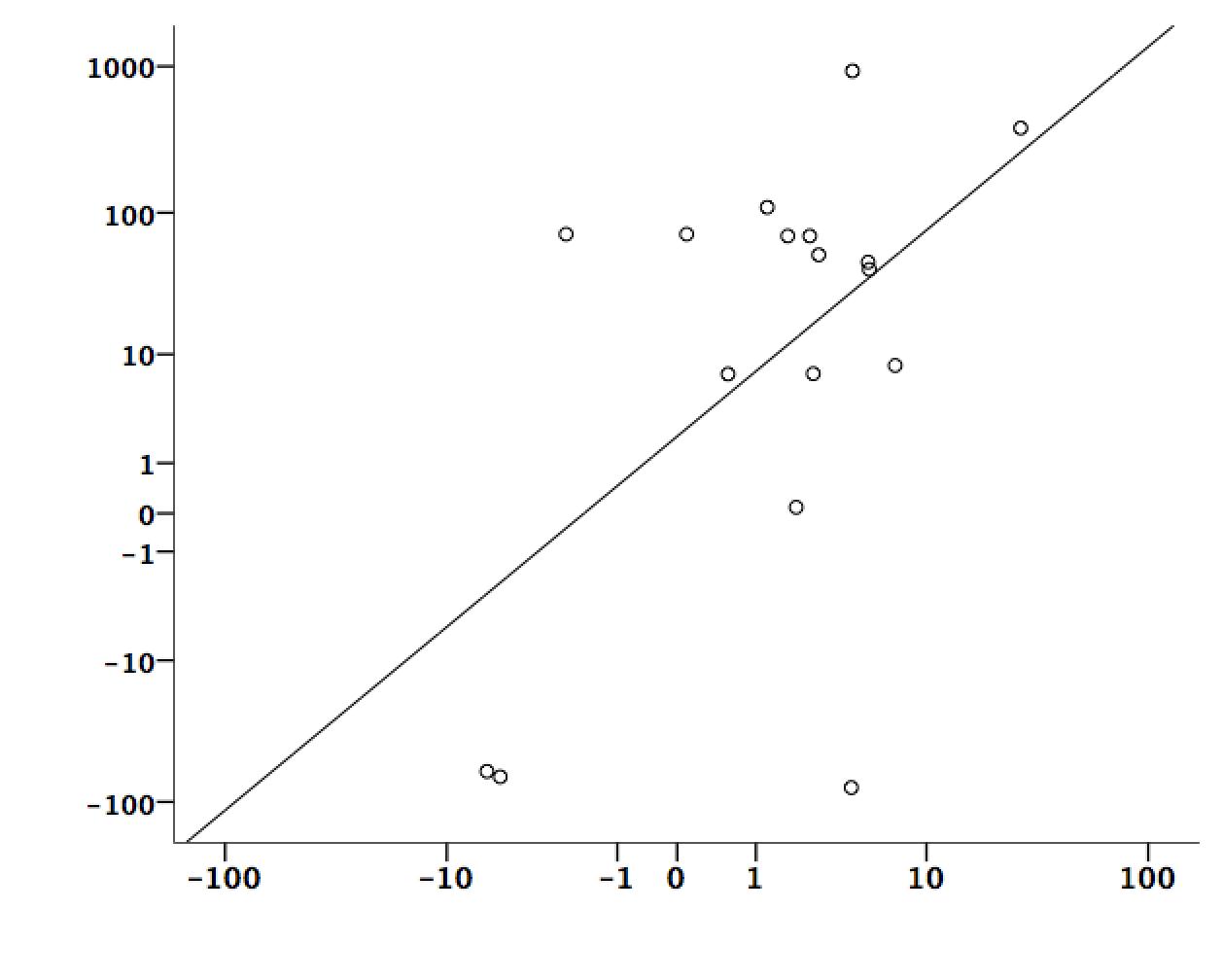


Log_10_ percent change in pre-meal ghrelin

Log_10_ percent change in BMI

r=0.82

p<0.001
