# Supplementary material for: The effect of H. pylori eradication on meal-associated changes in plasma ghrelin and leptin
Source: BMC Gastroenterol. 2011 Apr 14;11:37. doi: 10.1186/1471-230X-11-37 (PMC3089783; doi:10.1186/1471-230X-11-37)
Supplement: Additional file 3 — Supplemental tables. Provides information regarding the indications for upper GI endoscopy among study subjects (Table S1), findings during upper endoscopy (Table S2), Histologic score at three gastric sites according to H. pylori status (Table S3), levels of eight hormones related to energy homeostasis at the baseline evaluation of subjects according to H. pylori status and in relation to the test meal (Table S4), test-meal induced change and normalized change in hormone profile according to H. pylori status at baseline (Table S5), comparison of baseline and post-eradication meal-associated ghrelin profile in 21 originally H. pylori-positive subjects according to severity of baseline histologic gastritis (Table S6), and meal-associated changes in ghrelin profile in 28 subjectsa who had follow-up evaluation according to anatomical distribution of histologic gastritis at baseline (Table S7). [file 1471-230X-11-37-S3.DOC]

**Table S1.** Indications for upper GI endoscopy among 92 study subjects.

|  | **Percent with indication^a^** | | |
| --- | --- | --- | --- |
| **Indication for endoscopy** | ***H. pylori-***  **negative**  **(N = 38)** | ***H. pylori-***  **indeterminate**  **(N = 10)** | ***H. pylori-***  **positive**  **(N = 44)** |
| Heme-positive stool | 30 | 0 | 39 |
| Persistent heartburn | 16 | 30 | 11 |
| Abdominal pain | 16 | 20 | 11 |
| Iron deficiency | 13 | 20 | 16 |
| Barrett’s follow-up | 5 | 10 | 5 |
| Dysphagia | 5 | 10 | 0 |
| Ulcer follow-up | 3 | 10 | 4 |
| Nausea & vomiting | 3 | 0 | 0 |
| Other | 9 | 0 | 14 |

^a^ No differences between the *H. pylori*-positive and *H. pylori*-negative were significant (P>0.05)

**Table S2.** Findings during upper GI endoscopy among 92 study subjects.

|  | **Percent with indication^a^** | | |
| --- | --- | --- | --- |
| **Indication for endoscopy** | ***H. pylori-***  **negative**  **(N = 38)** | ***H. pylori-***  **indeterminate**  **(N = 10)** | ***H. pylori-***  **positive**  **(N = 44)** |
| Hiatal hernia | 18 | 3 | 13 |
| Esophagitis | 6 | 1 | 6 |
| Barrett’s esophagus | 9 | 1 | 5 |
| Erythematous gastropathy | 21 | 10 | 30 |
| Gastric erosions | 2 | 0 | 1 |
| Gastric ulcer | 1 | 2 | 1 |
| Fundic polyps | 3 | 0 | 3 |

^a^ No differences between the *H. pylori*-positive and *H. pylori*-negative were significant (P>0.05)

**Table S3:** Histologic score at three gastric sites among 38 *H. pylori* negative and 44 *H. pylori* positive subjects^a^

| **Site** |  | **Histologic score (Mean ± SD)** | | *P value*^b^ |
| --- | --- | --- | --- | --- |
|  |  | *H. pylori*-negative  (n=38) | *H. pylori*-positive  (n=44) |  |
| Antrum | Active gastritis | 0 ± 0 | 0.68 ± 0.25 | 0.12 |
|  | Chronic active gastritis | 0.29 ± 0.17 | 0.82 ± 0.97 | **<0.001** |
|  | Intestinal metaplasia | 0.29 ± 0.17 | 0.19 ± 0.49 | 0.84 |
|  | Atrophy | 0.09 ± 0.3 | 0.35 ± 0.60 | 0.37 |
| Body | Active gastritis | 0 ± 0 | 0.12 ± 0.50 | 0.17 |
|  | Chronic active gastritis | 0.03 ± 0.17 | 0.48 ± 0.67 | **<0.001** |
|  | Intestinal metaplasia | 0 ± 0 | 0.07 ± 0.34 | 0.22 |
|  | Atrophy | 0 ± 0 | 0.35 ± 0.70 | 0.33 |
| Fundus | Active gastritis | 0 ± 0 | 0.10 ± 0.49 | 0.24 |
|  | Chronic active gastritis | 0 ± 0 | 0.58 ± 0.71 | **<0.001** |
|  | Intestinal metaplasia | 0 ± 0 | 0.02 ± 0.16 | 0.36 |
| **All sites** | **Active gastritis (total)** | 0.6 ± 0.34 | 2.1 ± 1.0 | **<0.001** |

^a^Scoring based on the updated Sydney system [16].

^b^Mann-Whitney U test.

**Table S4: Levels of eight hormones related to energy homeostasis at the baseline evaluation of 82 subjects according to *H. pylori* status, and in relation to the test meal.**

|  | **Median (IQR) hormone concentration (pg/ml), by baseline *H. pylori* status** | | | | | | **Comparison of**  ***H. pylori* negative and**  ***H. pylori-*positive subjects**  **(p-value)** | |
| --- | --- | --- | --- | --- | --- | --- | --- | --- |
|  | ***H. pylori*-negative (n=38)** | | | ***H. pylori*-positive (n=44)** | | |  |  |
| **Hormone** | **Pre-meal** | **Post-meal** | **p^a^** | **Pre-meal** | **Post-meal** | **p^a^** | **Pre-meal** | **Post-meal** |
| Amylin | 40  (14-66) | 58  (15-117) | **0.028** | 16  (14-76) | 40  (15-104) | **0.004** | 0.78 | 0.78 |
| Insulin | 285  (161-459) | 957  (390-1,993) | **<0.001** | 295  (139-460) | 952  (475-1,735) | **<0.001** | 0.84 | 0.84 |
| Ghrelin | 1,321  (23-3,306) | 783  (24-2,243) | **0.001** | 1,623  (8-3,504) | 248  (12-1,329) | **<0.001** | 0.91 | 0.85 |
| GIP | 17  (6-31) | 121  (31-234) | **<0.001** | 15  (8-30) | 130  (72-237) | **<0.001** | 0.69 | 0.69 |
| GLP-1 | 23  (9-70) | 29  (9-72) | 0.87 | 27  (9-82) | 18  (9-66) | 0.46 | 0.65 | 0.65 |
| Leptin | 2,190  (961-7,085) | 2,840  (1,210-6,135) | **0.003** | 4,260  (1,850-7,540) | 5,680  (1,875-9.537) | **0.001** | 0.14 | 0.17 |
| PP | 72  (39-117) | 135  (77-177) | **<0.001** | 64  (30-136) | 136  (69-209) | **<0.001** | 0.81 | 0.81 |
| PYY | 49  (33-78) | 74  (52-110) | **0.001** | 48  (27-72) | 68  (47-95) | **<0.001** | 0.64 | 0.64 |

^a^ Wilcoxon’s signed rank test comparing pre-meal and post-meal values within *H. pylori* groups

^b^ Mann-Whitney U test, comparing values for *H. pylori*-negative and *H. pylori*-positive subjects

**Table S5:** Test-meal induced change and normalized^a^ change in hormone profile in 82 subjects, according to *H. pylori* status at baseline.

|  | **Median (IQR) change in hormone concentration (pg/ml) and normalized change, according to *H. pylori* status** | | | | **p^b^** | |
| --- | --- | --- | --- | --- | --- | --- |
|  | ***H. pylori* negative (n=38)** | | ***H. pylori* positive (n=44)** | |  |  |
| **Hormone** | **∆ Pre-meal-Post-meal** | **∆ normalized**  **(%)** | **∆ Pre-meal-Post-meal** | **∆ normalized**  **(%)** | **∆ Pre-meal-Post-meal** | **∆ normalized** |
| Amylin | 0  (9, 19) | 0  (-15, 76) | 2  (0, 90) | 2  (0, 121) | 0.93 | 0.81 |
| Insulin | 465  (5, 1676) | 108  (1, 582) | 368  (63, 1587) | 116  (28, 610) | 0.54 | 0.30 |
| Ghrelin | 183  (1, 1480) | -20  (-39, -2) | 146  (2840, 0) | -24  (-74, 0) | 0.87 | 0.85 |
| GIP | 95  (1, 232) | 527  (3, 2085) | 88  (43, 178) | 510  (106, 1634) | 0.30 | 0.85 |
| GLP-1 | 0  (7, 21) | 0  (-33, 23) | 0  (5, 15) | 0  (-45, 7) | 0.75 | 0.43 |
| Leptin | 110  (27, 606) | 3  (-3, 14) | 325  (15, 2198) | 16  (-1, 42) | 0.34 | 0.30 |
| PP | 32  (2, 87) | 48  (1, 130) | 47  (15, 87) | 93  (52, 199) | 0.16 | 0.16 |
| PYY | 12  (1, 23) | 38  (1, 67) | 22  (0, 37) | 34  (0, 78) | 0.28 | 0.47 |

^a^ Normalized by the individual pre-meal values.

^b^ Mann-Whitney U*-*test comparing *H. pylori*-negative and *H. pylori*-positive subjects.

**Table S6:** Comparison of baseline and post-eradication meal-associated ghrelin profile in 21 originally *H. pylori*-positive subjects, according to severity of baseline histologic gastritis.

| **Meal status at baseline** | **Median [IQR] ghrelin concentration (pg/ml)** | | *P value*^c^ |
| --- | --- | --- | --- |
|  | Low active gastritis^b^  (n=7) | High active gastritis^b^  (n=14) |  |
| Pre-meal | 516  [7-1580] | 3173  [989-3899] | **0.04** |
| Post-meal | 80  [7-538] | 925  [183-1378] | 0.12 |
| Normalized % change^a^ | -8  [-47 – 0] | -67  [-74 – 3] | 0.47 |
| **Meal status post-eradication** |  | | |
| Pre-meal | 1342^d^  [11 - 5447] | 1877  [447 - 3726] | 0.62 |
| Post-meal | 1594^d^  [11 - 3236] | 1961  [435 - 3910] | 0.39 |
| Normalized % change^a^ | 0  [-35 - 0] | -19  [-28 - -1] | **0.02** |

^a^Normalized % change= ([Post-meal] –[Pre-meal])/[Pre-meal]*100.

^b^Summation of inflammation scores for all sites allowed for the categorization of low (<2) vs high (>2) active gastritis.

^c^Wilcoxon’s signed rank test.

^d^P<0.05; compared to baseline value

**Table S7:** Meal-associated changes in ghrelin profile in 28 subjects^a^ who had follow-up evaluation, according to anatomical distribution of histologic gastritis at baseline

| **Change from baseline to eradicated [normalized]** | **Median [IQR] percent change in ghrelin concentration from baseline to follow-up** | | | *P value*^d^ |
| --- | --- | --- | --- | --- |
|  | No gastritis  (n=10) | Antral gastritis  (n=6) | Pan-gastritis  (n=12) |  |
| Pre-meal^b^ | -98  [-99 – -16] | 109  [59 – 244] | 7  [-62 – 46] | **0.01** |
| Post-meal^b^ | -98  [-99 – -1] | 494  [240 – 1300] | 135  [-22 – 380] | **0.04** |
| Meal effect^c^ | -100  [-309 – -100] | 33  [24 – 143] | -145  [-167 – -62] | **0.02** |

^a^Includes 21 *H.pylori*-positive and 7 *H. pylori*-negative subjects

^b^Normalized % change= ((Eradicated - Baseline)/Baseline)*100

^c^Change in meal effect= Normalized eradicated meal-associated change – Normalized baseline meal-associated change

^d^Kruskal-Wallis test.
